# Development and validation of multiple linear regression models for predicting total hip arthroplasty acetabular prosthesis

**DOI:** 10.1186/s13018-024-04526-0

**Published:** 2024-01-17

**Authors:** Ze-hui Zou, Xu-qiang Liu, Wei-hua Li, Xin-tao Zhou, Xiao-feng Li

**Affiliations:** Department of Sports Medicine, Orthopedic Hospital, The First Affiliated Hospital, Jiangxi Medical College, Nanchang University, Nanchang, China

**Keywords:** Total hip arthroplasty, Multiple linear regression, Predictive modeling, Acetabular prosthesis, Anthropometric data

## Abstract

**Purpose:**

To establish a multivariate linear equation to predict the diameter (outer diameter) of the acetabular prosthesis used in total hip arthroplasty.

**Methods:**

A cohort of 258 individuals who underwent THA at our medical facility were included in this study. The independent variables encompassed the patients' height, weight, foot length, gender, age, and surgical access. The dependent variable in this study was the diameter of the acetabular prosthesis utilized during the surgical procedure. The entire cohort dataset was randomly partitioned into a training cohort and a validation cohort, with a ratio of 7:3, employing the SPSS 26.0 software. Pearson correlation analysis was conducted to examine the relationships between the patients' height, weight, foot length, gender, age, surgical access, and the diameter of the acetabular prosthesis in the training cohort. Additionally, a multiple linear regression equation was developed using the independent variables from the training cohort and the diameter of the acetabular prosthesis as the dependent variable. This equation aimed to predict the diameter of the acetabular prosthesis based on the patients' characteristics. The accuracy of the equation was evaluated by substituting the data of the validation cohort into the multiple linear equation. The predicted acetabular prosthesis diameters were then compared with the actual diameters used in the operation.

**Results:**

The correlation analysis conducted on the training cohort revealed that surgical access (*r* = 0.054) and age (*r* = −0.120) exhibited no significant correlation with the diameter of the acetabular prosthesis utilized during the intraoperative procedure. Conversely, height (*r* = 0.687), weight (*r* = 0.654), foot length (*r* = 0.687), and sex (*r* = 0.354) demonstrated a significant correlation with the diameter of the acetabular prosthesis used intraoperatively. Furthermore, a predictive equation, denoted as Y (acetabular prosthesis diameter in mm) = 20.592 + 0.548 × foot length (cm) + 0.083 × height (cm) + 0.077 × weight (kg), was derived. This equation accurately predicted the diameter within one size with an accuracy rate of 64.94% and within two sizes with an accuracy rate of 94.81%.

**Conclusion:**

Anthropometric data can accurately predict the diameter of acetabular prosthesis during total hip arthroplasty.

## Introduction

Total hip arthroplasty (THA) has emerged as a highly efficacious surgical intervention in the twenty-first century, effectively alleviating pain and restoring hip mobility in patients with advanced hip ailments [1, 2]. Given the escalating aging demographic, the demand for THA within a specific age bracket is anticipated to rise [3, 4]. In a healthcare landscape that emphasizes value-based care and diminishing reimbursement, the provision of efficient, timely, and cost-effective patient care assumes paramount importance [5]. Effective preoperative planning and predictive modeling have the potential to mitigate surgical complications and enhance the efficacy of implant providers and operating room procedures [6]. The existing preoperative planning process for THA entails conducting a 3D CT thin-layer scan of both hips, segmenting patient data into transverse, coronal, and sagittal planes, and subsequently fine segmenting the image for bilateral 3D stereo modeling [7]. However, this approach not only imposes a financial burden on patients but also escalates the overall cost of healthcare [8]. In contrast, anthropometric measurements, including height, weight, and foot length, are easily obtainable and can be conducted prior to surgical procedures. Murphy et al. [9] have presented a model for predicting prosthesis selection in THA based on demographic data, demonstrating some degree of accuracy. However, studies investigating the prediction of acetabular prosthesis models remain limited, according to the authors' knowledge. Rehman et al. [10] have reported a significant positive correlation between shoe size and knee implant size in all knee replacement surgeries, enabling surgeons to confidently determine implant size without the need for preoperative planning. In this study, the inclusion of foot length in the THA can reduce the error associated with shoe size and establish a more accurate prediction model.

## Materials and methods

### Inclusion and exclusion criteria

The inclusion criteria for this study encompassed individuals who met the following conditions: (1) undergoing their first total hip replacement procedure, (2) experiencing an uncomplicated total hip replacement, (3) denying any prior foot surgery, and (4) exhibiting no alteration in foot length from adulthood to the preoperative period. Conversely, the exclusion criteria involved individuals who were (1) undergoing hip revision surgery, (2) receiving an initial replacement of a complex hip, (3) having a history of foot surgery, and (4) displaying a change in foot length from adulthood to the preoperative period.

### General information

A total of 258 patients who underwent total hip arthroplasty at our hospital between March 2019 and November 2022 were selected for inclusion in this study. Of these cases, 34 utilized the Direct Anterior Approach (DAA) while 224 utilized the Posterolateral Approach (PLA), with 75 presenting femoral neck fractures, 7 intertrochanteric fractures, 14 exhibiting developmental hip dysplasia, and 162 displaying necrosis of the femoral head. These diagnoses were confirmed through imaging reports, and subsequently, all patients underwent total hip arthroplasty. The study population consisted of 119 males and 139 females, ranging in age from 17 to 93 years with a mean age of 63.80 ± 16.53 years (Table 1). Prior to surgery, all patients provided informed consent and the study received approval from the hospital's Ethics Committee.

**Table 1:** The general information of whole study cohort (training and testing)

Variable	Whole study cohort (*n* = 258)
Mean age, y (SD)	63.80 (16.53)
Surgical approach, n (%)	
DAA	34 (13.18)
PLA	224 (86.82)
Sex, n (%)	
Male	119 (46.12)
Female	139 (53.88)
Primary diagnosis, n (%)	
Femoral neck fracture	75 (29.07)
Intertrochanteric fracture	7 (2.71)
Developmental hip dysplasia	14 (5.43)
Necrosis of the femoral head	162 (62.79)

### Parameter collection

A total of 258 patients who underwent primary total hip arthroplasty at the First Affiliated Hospital of Nanchang University were included in this study. Prior to surgery, the patients' height, weight, and foot length were measured. The height and weight measurements were conducted using the same measuring instrument. Foot length was defined as the maximum distance from the back of the heel to the tip of the longest toe (either the first or second toe) [11]. The patients' barefoot foot length was measured using a foot length measuring device. Additionally, baseline information including the patient's gender and age was collected.

### Surgical methods

#### DAA group

The anesthesia administration is deemed effective as the patient assumes a supine position. Subsequently, an anterolateral incision is made on the affected hip, systematically dissecting the skin, subcutaneous tissue, and superficial fascia. The broad fascia is then incised, allowing superficial access through the gap between the broad fascia tensor and the suture muscle. Further deep access is achieved through the gap between the rectus femoris muscle and the lateral femoral muscle. A T-shaped incision is made on the joint capsule, thereby exposing the femoral head and neck. Finally, a saw is employed to perform the bone resection. The acetabular file is utilized to grind and file the acetabular cartilage until the articulating surface exposes the cancellous bone, resulting in visible bleeding. Subsequently, an appropriate acetabular prosthesis is inserted into the acetabulum. The femur is then expanded, and upon achieving satisfactory expansion, the femoral stem of the prosthesis is implanted. The head of the femur is also installed, followed by the resetting of the hip joint. The hip joint's stability and tightness are assessed, and tranexamic acid is used for irrigation. A drain is inserted, and the incision is meticulously closed in layers.

#### PLA group

The anesthesia is administered successfully, and the patient is positioned in a healthy side lying posture with the affected hip facing upward. The posterior lateral incision is made, systematically cutting through the layers of skin, subcutaneous tissues, and superficial fascia. The broad fascia is then opened, followed by the removal of the gluteus maximus femoral stop, pyriformis muscle, and upper and lower twins. The inner muscle attachment point of the greater trochanter is closed, exposing the affected side of the hip joint. Finally, the bone is cut using a pendulum saw. The acetabular file is used to sequentially grind and file the joint surface of the acetabulum until the cancellous bone is exposed and obvious bleeding occurs. Following this, a suitable acetabular prosthesis is installed. The femur is then sequentially expanded to accommodate the prosthesis femoral stem, and the head of the femur is installed. The hip is reset and the joint surface is examined for loose bone and obvious bleeding. This process is repeated until the joint surface is fully exposed to the cancellous bone and obvious bleeding occurs, at which point the appropriate acetabular prosthesis is installed and the femur is expanded accordingly. After achieving satisfaction, proceed with the installation of the prosthesis femoral stem, followed by the installation of the femur head and resetting of the hip joint. Subsequently, ensure the tightness of the components and perform aminomethylenic acid irrigation. Place a drain and proceed to suture the incision layer by layer.

### Statistical analysis

Statistical analysis was conducted using SPSS 26.0 software. Measurement data were presented as (*X* ± s), while count data were expressed as frequency. Pearson's correlation analysis was performed on various variables including height, weight, gender, foot length, surgical access, age, and acetabular prosthesis diameter. The entire patient cohort was randomly divided into a training cohort and a validation cohort in a 7:3 ratio using SPSS. The independent variables in this study included the height (cm), weight (kg), foot length (cm), gender, and age of the patients. The dependent variable was the diameter of the acetabular prosthesis (mm). Multivariate stepwise linear regression was performed using the Stepwise method, with a selection criterion of *p* value < 0.05 and a removal criterion of *p* value > 0.10. This analysis resulted in a multivariate linear equation incorporating foot length, height, and weight as independent variables. The equation demonstrated the highest goodness-of-fit and was subsequently employed in a validation cohort to assess the accuracy of the predictive model for various implant sizes.

## Results

A unidirectional correlation analysis was conducted to examine the relationship between body parameters and acetabular size. Pearson's correlation analysis revealed that surgical access (*r* = 0.054) and age (*r* = −0.120) exhibited no significant correlation with the diameter of the acetabular prosthesis utilized in the surgical procedure, as indicated by a *p* value > 0.05. Conversely, height (*r* = 0.687), weight (*r* = 0.654), foot length (*r* = 0.687), and sex (*r* = 0.354) demonstrated a significant correlation with the diameter of the acetabular prosthesis employed in the operation, with a *p* value < 0.05 (Table 2).

**Table 2:** Results of Pearson's correlation analysis between human parameters and acetabular dimensions

Human parameters	*r* value	*p* value
(A person's) height	0.687	< 0.001
Weight	0.654	< 0.001
Distinguishing between the sexes	0.354	< 0.001
Length of foot	0.687	< 0.001
(A person's) age	− 0.120	0.108
Surgical access	0.054	0.474

### Establishment of multiple regression and prediction

Multiple stepwise regression analysis was conducted to determine the relationship between anthropometric parameters and acetabular dimensions. The dependent variable in this analysis was the diameter of the acetabular prosthesis, while other factors were considered as independent variables. The stepwise method was employed, with a significance level of *p* value < 0.05 for variable selection and *p* value > 0.10 for variable removal. In the initial step of the regression analysis, the variable "foot length" was chosen as the dependent variable, exhibiting a composite correlation coefficient of *R* = 0.687. The initial step involved selecting the independent variable "foot length" and yielded a compound correlation coefficient of *R* = 0.687. Subsequently, the independent variable "body weight" was chosen in the second step, resulting in an *R* value of 0.745. In the third step, the independent variable "height" was once again selected, yielding an *R* value of 0.759. The findings of the multivariate linear step-by-step regression analysis can be found in Table 2. The multiple linear stepwise regression equation is as follows: Y (acetabular cup prosthesis size diameter in mm) = 20.592 + 0.548 × foot length (cm) + 0.083 × height (cm) + 0.077 × weight (k)g) (Table 3).

**Table 3:** Multiple stepwise regression analysis of acetabular dimensions with anthropometric parameters

Independent variable	Regression coefficient B	Standard error (SE)	Standardized regression coefficient	*t* value	*p* value
Constant term (math.)	20.592	2.904	–	7.092	< 0.001
Length of foot	0.548	0.143	0.307	3.822	< 0.001
(A person's) height	0.083	0.027	0.253	3.018	0.003
Weight	0.077	0.018	0.293	4.354	< 0.001

### Conformity between predicted and actual acetabulum

The multiple linear stepwise regression equations predicted 63.94% accuracy within one size and 94.81% accuracy within two sizes (Table 4).

**Table 4:** The coincidence rate between predicted acetabulum and actual acetabulum

Difference between predicted and actual values (mm)	± 0	± 1	± 2	± 3	± 4	≥ ± 4
Accuracy of predicting acetabular prosthesis size	20.78%	19.48%	24.68%	11.69%	18.18%	5.19%

### Compliance of predicted acetabulum with preoperatively planned acetabulum

Fifty-seven patients who underwent preoperative planning were selected and a formula was used to predict the diameter of the acetabular prosthesis to be used intraoperatively, which was compared to the preoperative planning carried out to derive an accuracy rate (Table 5).

**Table 5:** Compliance rate between predicted acetabulum and planned acetabulum

Difference between projected and planned values	± 0	± 1	± 2	± 3	± 4	> ± 4
Accuracy	47.37%	29.82%	15.79%	5.26%	0	1.75%

## Discussion

Anthropometric variables such as height, weight, and foot length have been identified as potential indicators of the size of acetabular prostheses used in THA. Previous research has demonstrated a correlation between shoe size, height, weight, gender, and age with the size of knee and hip prostheses [12, 13]. However, it is worth noting that no study to date has incorporated foot length as a variable in the prediction model. By including foot length in this study, the potential for error associated with shoe size can be reduced, thereby enhancing the accuracy of the prediction model. In this study, the coefficient of influence analysis revealed that foot length exhibited the highest magnitude of impact on the size of the acetabular prosthesis employed in surgical procedures, followed by height and weight. This finding underscores the significance of foot length as the primary determinant of acetabular prosthesis size. Previous forensic research has demonstrated the utility of foot length and shoe size in predicting the gender and height of victims, thereby highlighting the role of foot length as a reliable indicator of skeletal structure within the human body. Consequently, foot length can be considered a representative measure of the overall skeletal framework, albeit to a limited extent [14–18]. The patient's foot length, height, and weight can be easily measured, and these measurements can be used in a formula to predict the size of the acetabular prosthesis that will be utilized in the operation. This preoperative planning can alleviate the workload of the healthcare provider and enhance the efficiency of the operating room to some extent. In this study, the size of the acetabular prosthesis is standardized using the outer diameter (mm) to account for variations among multiple manufacturers, thereby making the prediction equation more universally applicable. The application of this approach is applicable to any supplier of implants. In the past, preoperative planning relied on bilateral hip X-rays and hip CT scans, followed by the utilization of specialized preoperative planning software [19, 20]. However, this method inevitably imposes a financial burden on the patient and increases the workload of the hospital. Alternatively, a simpler approach utilizing anthropometric data can yield equally effective preoperative plans [21]. Moreover, the coefficient of determination *R*^2^, an essential metric in multiple linear regression analysis, serves as an indicator to assess the adequacy of multiple linear regression models [22]. In this study, the obtained *R*^2^ value of 0.576 signifies that approximately 57.6% of the variation in acetabular cup diameter can be accounted for by changes in height, weight, and foot length. It is worth noting that *R*^2^ ranges from 0 to 1, and the fact that the *R*^2^ value in this study is 0.576 implies the existence of unexplained variability. There are multiple factors that can account for this phenomenon. Firstly, the selection criteria for determining the appropriate size of the fitted acetabular cup in this study were based on specific parameters. These parameters included the placement of the intraoperative acetabular cup at an angle of 30°–45° of abduction and 15°–20° of anterior tilt. Additionally, the acetabulum was meticulously polished using an acetabular file until it reached the subchondral hemorrhage. Furthermore, the horseshoe fossa was ground down to achieve a flat level, and the thickness of the anterior and posterior acetabular wall was adjusted to a moderate level. These steps were taken to ultimately determine the appropriate size of the fitted acetabular prosthesis [23, 24]. This study also incorporated multiple surgeons due to the recognition that while the criteria for placing the acetabular prosthesis remain consistent among surgeons, their individual surgical techniques and subjective perspectives may introduce potential interference in achieving a well-fitted acetabular prosthesis. Consequently, this factor may contribute to the diminished accuracy of the equation. Nonetheless, the inclusion of multiple surgeons enhances the applicability and generalizability of the findings. Secondly, variations in disease types can also impact the dimensions of the acetabular prosthesis inserted during surgery. For instance, in cases of femoral head necrosis and femoral neck fracture, the acetabulum affected by femoral head necrosis may exhibit deformation, whereas the acetabulum affected by femoral neck fracture typically does not. To illustrate, if two patients with identical foot length, height, weight, and gender experience either femoral neck fracture or femoral head necrosis, the actual size of the implanted acetabular prosthesis may not be identical. Consequently, future improvements are warranted in this regard. Hence, future advancements may involve the development of more accurate predictive models encompassing various disease types. This study further incorporated patients with preoperative planning and compared it to our prediction formula, revealing a compliance rate of 92.99% within a single size. Consequently, in certain remote healthcare facilities where preoperative planning is not feasible, our straightforward preoperative planning approach can be employed to yield improved prediction outcomes.

## Limitation

Nevertheless, it is important to acknowledge the limitations of this study, namely the small sample size and the fact that it was conducted at a single center. To enhance the validity and generalizability of future research, it is recommended to incorporate a larger sample size and conduct a multi-center study. By doing so, a more precise predictive model can be established. Nonetheless, this study contributes to the field by presenting a methodology and demonstrating that anthropometric data can effectively forecast the size of the acetabular cup utilized THA.

## Conclusion

The utilization of patient anthropometric measurements such as foot length, height, and weight can effectively forecast the dimensions of the acetabular prosthesis in hip arthroplasty through the development of a multiple linear regression equation. This approach has the potential to enhance the efficiency of the implant supplier and the operating room procedure, while also mitigating the potential financial strain to some extent.

## Data Availability

The datasets used during the current study are available from the corresponding author on reasonable request.
